# Assessing and removing the effect of unwanted technical variations in microbiome data

**DOI:** 10.1038/s41598-022-26141-x

**Published:** 2022-12-23

**Authors:** Muhamad Fachrul, Guillaume Méric, Michael Inouye, Sünje Johanna Pamp, Agus Salim

**Affiliations:** 1 Cambridge Baker Systems Genomics Initiative, Baker Heart and Diabetes Institute, Melbourne, VIC 3004 Australia; 2 Department of Clinical Pathology, University of Melbourne, Parkville, VIC 3010 Australia; 3 Department of Infectious Diseases, Central Clinical School, Monash University, Melbourne, VIC 3004 Australia; 4 Cambridge Baker Systems Genomics Initiative, Department of Public Health and Primary Care, University of Cambridge, Cambridge, UK; 5 British Heart Foundation Cardiovascular Epidemiology Unit, Department of Public Health and Primary Care, University of Cambridge, Cambridge, UK; 6 British Heart Foundation Centre of Research Excellence, University of Cambridge, Cambridge, UK; 7 Health Data Research UK Cambridge, Wellcome Genome Campus and University of Cambridge, Cambridge, UK; 8 National Food Institute, Technical University of Denmark, 2800 Kgs. Lyngby, Denmark; 9 Novo Nordisk Foundation Center for Biosustainability, Technical University of Denmark, 2800 Kgs. Lyngby, Denmark; 10 Centre for Epidemiology and Biostatistics, Melbourne School of Population and Global Health, The University of Melbourne, Melbourne, VIC 3010 Australia; 11 School of Mathematics and Statistics, The University of Melbourne, Melbourne, VIC 3010 Australia; 12 Department of Population Health, Baker Heart and Diabetes Institute, Melbourne, VIC 3004 Australia; 13 Department Mathematics and Statistics, La Trobe University, Bundoora, VIC 3086 Australia

**Keywords:** Computational biology and bioinformatics, Data processing, Quality control, Statistical methods, Metagenomics, Microbiome

## Abstract

Varying technologies and experimental approaches used in microbiome studies often lead to irreproducible results due to unwanted technical variations. Such variations, often unaccounted for and of unknown source, may interfere with true biological signals, resulting in misleading biological conclusions. In this work, we aim to characterize the major sources of technical variations in microbiome data and demonstrate how in-silico approaches can minimize their impact. We analyzed 184 pig faecal metagenomes encompassing 21 specific combinations of deliberately introduced factors of technical and biological variations. Using the novel Removing Unwanted Variations-III-Negative Binomial (RUV-III-NB), we identified several known experimental factors, specifically storage conditions and freeze–thaw cycles, as likely major sources of unwanted variation in metagenomes. We also observed that these unwanted technical variations do not affect taxa uniformly, with freezing samples affecting taxa of class *Bacteroidia* the most, for example. Additionally, we benchmarked the performances of different correction methods, including ComBat, ComBat-seq, RUVg, RUVs, and RUV-III-NB. While RUV-III-NB performed consistently robust across our sensitivity and specificity metrics, most other methods did not remove unwanted variations optimally. Our analyses suggest that a careful consideration of possible technical confounders is critical during experimental design of microbiome studies, and that the inclusion of technical replicates is necessary to efficiently remove unwanted variations computationally.

## Introduction

The technological advances in sequencing technologies have made microbiome studies more accessible and meaningful. From amplifying short 16S rRNA hypervariable regions to taking advantage of long-read sequencing, the breadth of data options has enabled the field to flourish in the past couple of decades, allowing a better understanding of the role of microbiomes in numerous ecological, environmental and clinical contexts^1^. For example, the dynamics of the gut microbiome is now known to be influenced by environment and diet, and perturbations (or “dysbiosis”) have been linked to chronic conditions such as cardiometabolic diseases and type 2 diabetes (T2D)^2–4^. As a result, the human microbiome is now considered an important biological basis for potential therapeutic targets^5,6^. However, the accelerated growth of microbiome studies using a very wide range of technological methods and experimental designs comes with a cost on reproducibility. Any valuable application from microbiome studies could be largely hindered by the lack of reproducibility due to the presence of unwanted technical variations^7,8^.

Microbiome studies can differ considerably when it comes to the experimental approaches; and each step of the workflow, including variations introduced by the experimenter, has the potential to introduce artificial results due to unwanted technical variations^9,10^. For example, under-sampling might occur due to the lack of consideration during initial collection process, resulting in zero reads detected for certain microbiota that actually exist in the environment due to their underrepresentation in the samples^11^. Additionally, specific stool collection kits have been shown to impact microbial abundance of faecal samples differently^12^. DNA extraction, library preparation kits, storage condition, storage time, and choice of sequencing platforms have also been found to introduce artificial variations in microbial abundance^9,10,13,14^. Contamination of external microbial taxa can also contribute to unwanted variations and can happen at any stage of the experiment. For instance, recent studies have found that some library preparation kits used before sequencing could introduce specific microbial taxa coming from reagents in the kit^15^. This issue is observed to be even more critical in low-biomass samples, even leading to debated interpretations on the existence of a microbiome in environments that might not have any, such as the human placenta or the meconium^15–18^. Left unaddressed, such unwanted variations can considerably confound true biological signals and result in misleading conclusions.

Various computational methods have been developed to correct for the presence of unwanted effects within and between experiments. Popular methods such as ComBat^19^ and RUV (Removing Unwanted Variations)^20^, originally developed for transcriptomics datasets, have also been considered for microbiome studies^9,21^ but do not particularly take into account the specific characteristics of microbiome data such as compositionality. Indeed, contrary to features in transcriptomics datasets, the presence of each taxa is not independent from the rest of the taxa in the microbiome, and raw abundance information acquired after sequencing is not directly representative of the actual abundance in the environment due to the limit of the sequencing depth that each platform has^22^, a problem that is not commonly addressed in current studies^23^. Additionally, microbiome data are typically very sparse, as many features are present in only a very few samples. Log-transformation of raw abundances, a process allowing for more robust statistical analyses to be performed, prerequires to substitute zero-values with a constant arbitrary number, also known as “pseudocount”. Despite being part of the compositional data analysis (CoDA) standards, there is an argument against adding pseudocount then using log-transformation in analyzing highly sparse datasets in the form of counts per sample, as it changes the ratio of taxa abundance substantially, diminishes variance from less abundant taxonomic groups and artificially exaggerates the differences between zero and non-zero values^24,25^.

In this study, we aim to characterize the major sources of technical variations in microbiome data and demonstrate how a state-of-the art approach can minimize their impact on downstream analyses. Using a dataset containing 184 faecal microbiome samples from pigs comprising up to 21 unique combinations of technical variations, we used RUV-III-NB^26^, a robust batch correction tool which utilizes Negative Binomial (NB) distribution to estimate and adjust for unwanted variations without the need to add prior pseudocounts, to identify parts of the experimental workflow that introduce critical unwanted variations that affect observed microbial abundances. We then compare the performance of RUV-III-NB to other popular tools including ComBat, ComBat-Seq, RUVg, and RUVs and demonstrate the comparative advantage of RUV-III-NB in both retaining biological signals and removing unwanted effects.

## Results

**Figure 1:**
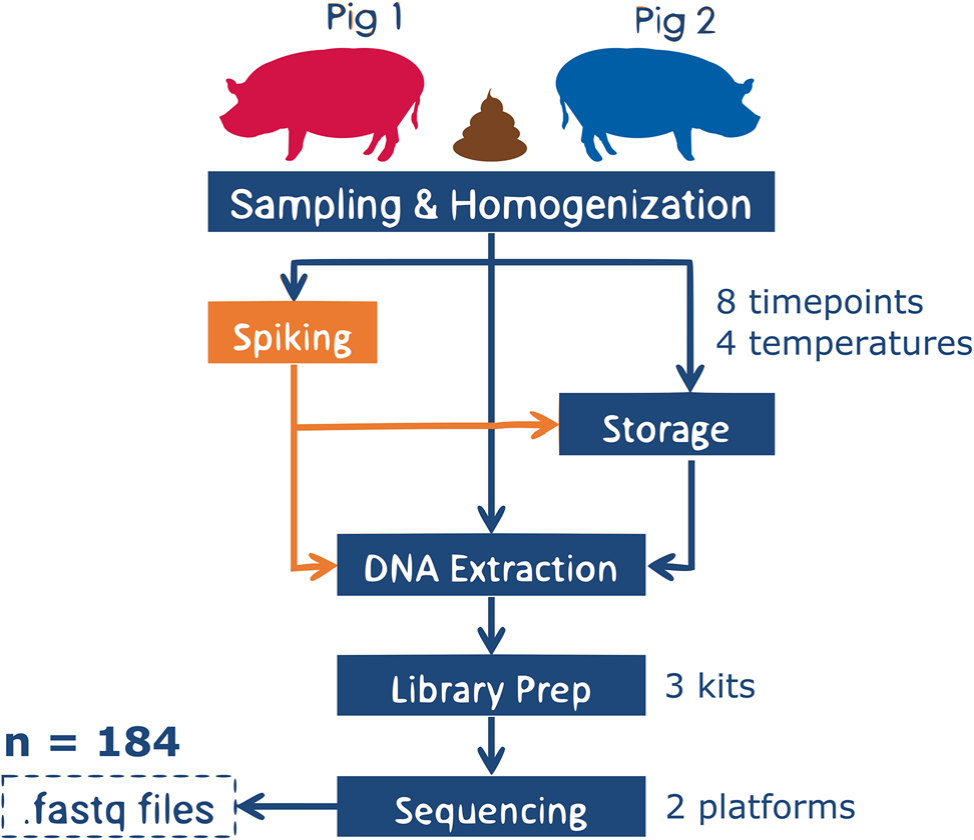
Experimental workflow of the dataset, showing the sources of possible unwanted variations, some of which correlated with the calculated unwanted factors.

Faecal samples were taken from 2 pigs and for each pig, half of the aliquots were spiked with live microbial cells, 6 of which bacterial and 2 eukaryotic. Details on the quantities of and how the spike-ins were added are described in the Supplementary Materials of a previous publication that describes the experiment^27^. Both the spiked and unspiked samples were subjected to different experimental factors, namely storage conditions (temperature, storage length), DNA extraction methods, and library preparations (Fig. 1). The spiked samples were sequenced using HiSeq platform, while the unspiked samples were sequenced using NextSeq and HiSeq platforms. Sequencing depth ranged from 2,489,951 to 11,852,857 reads, with an average of 5,675,095 reads. Among spiked samples, sequencing depth averaged 6,020,192 reads. In this paper, unless otherwise stated, we are focusing our analyses on the spiked samples only, thus we could not examine the role of sequencing platforms in introducing unwanted variations because all spiked samples were sequenced using the same platform, namely HiSeq.

### Unwanted technical variations exist in microbiome data, and CLR transformation alone is not effective

**Figure 2:**
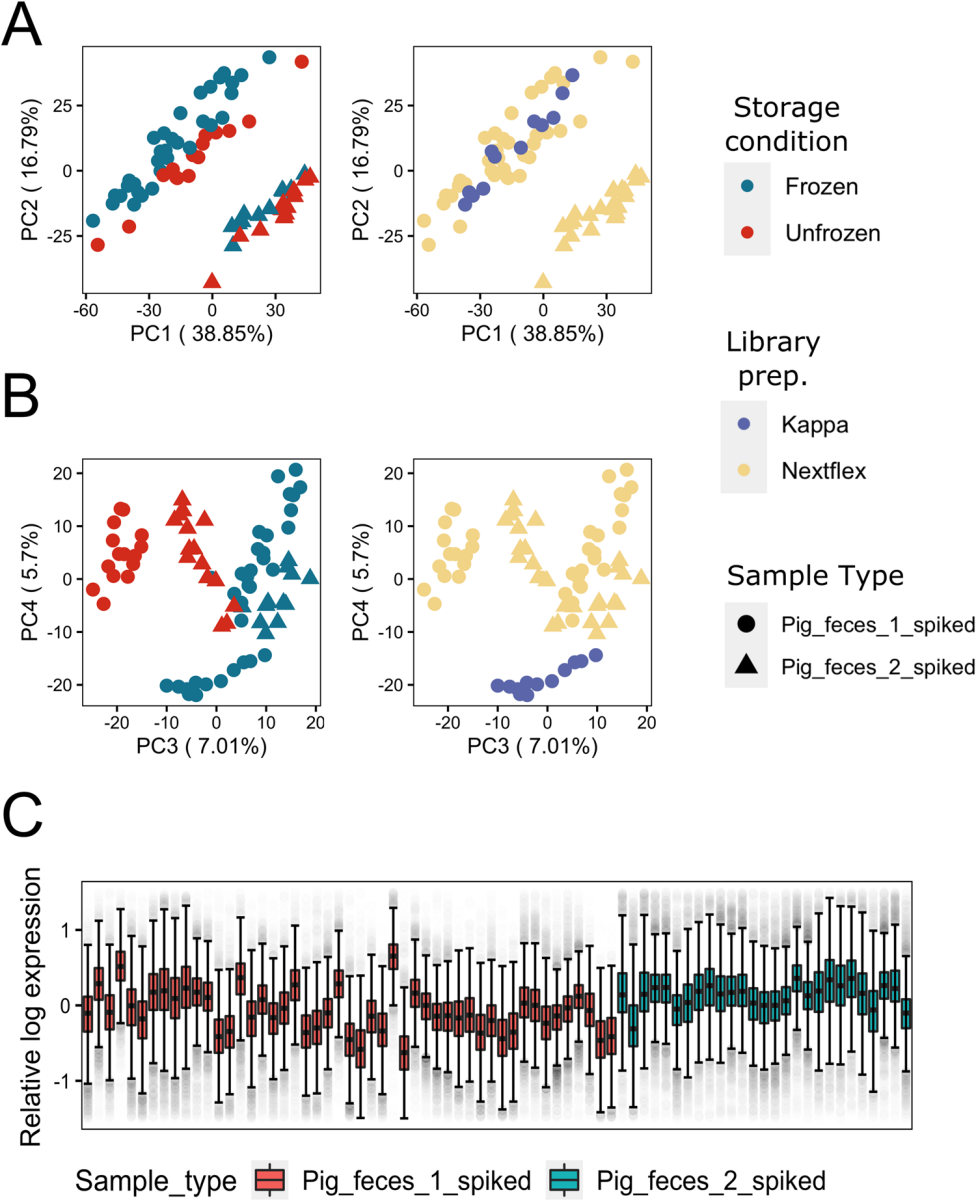
Visualizations of the CLR-normalized data of all spiked samples: **(A)** Principal component analysis (PCA) plots showing sample clustering based on sample origin (pig1 vs pig 2) explained by PC1 and PC2, while **(B)** separation of samples based on storage conditions and library preparation kits used were explained by PC3 and PC4, respectively. **(C)** Relative log expression (RLE) plot of all spiked samples, showing considerable variations based on deviation of the medians between samples of the same source, as well as dissimilarity of interquartile ranges (IQR)**.**

We performed principal component analysis (PCA) on the spiked pig metagenomes to assess the extent of different experimental factors in introducing unwanted variations in the data. Aitchison’s centered log-ratio (CLR) transformation was applied prior to PCA as a recommended normalization step for compositional data^23^. If experimental factors introduce little-to-no unwanted variations, we expect that the first few PCs capture between-pig biological variations, as is evident in PC1 and PC2 (Fig. 2A). Yet, PC3 and PC4 of the CLR-normalized data (Fig. 2B) reveal clustering of samples based on storage conditions and the library preparation kit. This clustering is also reflected by the silhouette scores based on the top 4 PCs (*ss* = 0.488). We also used relative log expression (RLE) plot^28^ of the CLR-normalized data to assess overall unwanted variations that exist within a dataset. Without unwanted variations, we expect little-to-no variations in the median (center of the box) and interquartile range (length of the box) of RLE for samples belonging to the same pig. Yet in this case, the RLE plot showed considerable variabilities in median and interquartile range between samples from the same pig **(**Ω_*RLE*_ score = 3.98; Fig. 2C), further confirming the presence of unwanted technical variations within the data which could not be corrected through CLR transformation.

### RUV-based methods remove unwanted variations better than ComBat-based methods

Having established the existence of unwanted variations in our data, we then compared the performance of three RUV-based methods (RUV-III-NB^26^, RUVg^20^ and RUVs^20^) and two ComBat-based methods (ComBat^19,29^ and ComBat-seq^30^) in removing unwanted variations and retaining biological variations. Both RUV-based and ComBat-based methods require users to provide replicate matrix that contains information about which samples belong to which pig, but as RUV-based methods do not assume users have complete knowledge of the underlying factors causing the unwanted variations, they require users to supply a list of *negative control taxa* whose variations will be used to estimate the unknown unwanted factors (see *RUV-III-NB batch correction* section in Methods for details). We use three different versions of negative control taxa: (1) spike-in taxa, (2) empirical negative control taxa and (3) combination of spike-ins and empirical negative control taxa. However, for the purpose of comparing methods, we simply average the performance metric of a RUV-based methods over different versions. The impact and consequences of choosing different negative control taxa are detailed in the fourth section of the results (*Supplementing control features with empirical negative control taxa are preferred when using RUV-based methods*) below.

**Figure 3:**
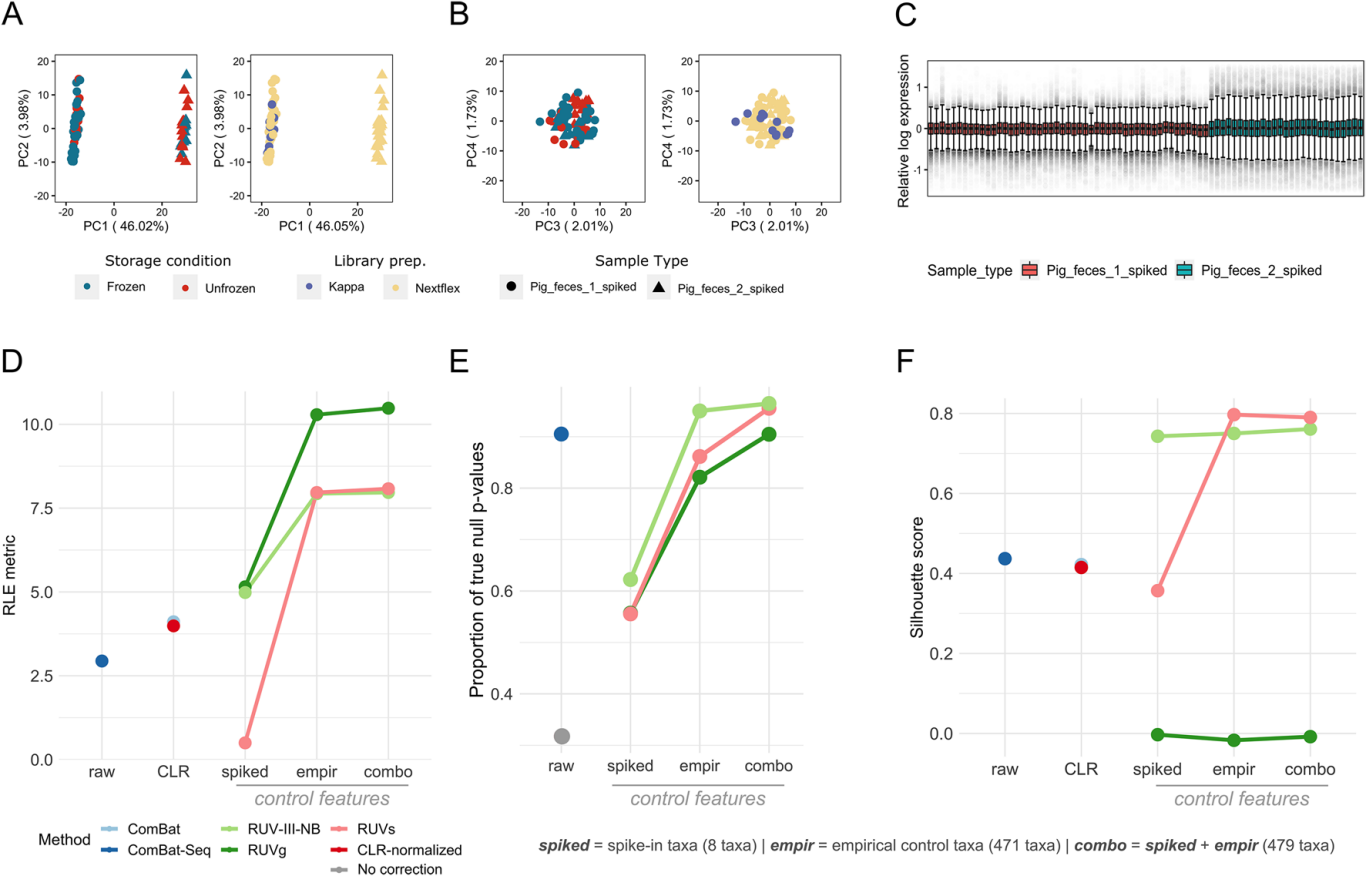
**(A)** PC1 and PC2 of all spiked samples after RUV-III-NB correction showing retained separation based on sample origin, yet **(B)** clustering based on storage conditions and library preparation kits used is no longer present in PC3 and PC4, suggesting successful removal of unwanted variations. **(C)** RLE plot of all spiked samples after RUV-III-NB correction, showing successful removal of unwanted variations represented by the medians of sample from the same individual being as close to zero as possible and as linear to each other as possible, as well as similar interquartile ranges (IQR) between the samples visualized by the size of the boxplot. **(D)** Comparison of correction method performances between mere CLR-normalization, ComBat-based methods (which only processes either raw or CLR-transformed dat), and RUV-based methods (which leverages negative control features); based on relative log expression (RLE metric), where higher number indicates better removal of overall unwanted variations. With the exception of RUVs with solely spike-in taxa as control features, RUV methods in average performed better compared than ComBat-based methods. **(E)** Proportion of true null p-values (*π*_*0*_) of samples with no correction and samples corrected using different methods after differential abundance analysis. Since storage conditions were found to be the main batch variable in this dataset, comparison was done between frozen and unfrozen samples of samples from the same source (pig 1). Since edgeR requires integer counts as input, ComBat was omitted from this comparison. RUV-III-NB resulted in the highest pi0 overall when using the combination set of control taxa, and still performed better than ComBat-seq when using solely empirical taxa as control features. **(F)** Comparison of correction method performances in preserving individual biological information through silhouette scores, which are calculated based on the main PCs of spiked samples and explain how well samples separate between defined groups (higher = better). RUVs using solely empirical control features had the highest silhouette score in separating pig 1 and pig 2 samples, yet correction using RUV-III-NB had the highest average silhouette score overall—indicating its consistency despite the different set of control features. RUVg performed really poorly overall, with silhouette scores using all the different control features placing significantly lower than even CLR-normalized data.

To assess how well each method removes the unwanted variation, we deployed several approaches. First, we performed PCA on the microbiome composition matrix data after each correction method and calculated the average width of the silhouette score statistics (ss) for clustering by storage conditions, which seemed to be the main source of unwanted variations based on clustering of CLR-normalized samples (Fig. 2B) using the top four principal components (PCs). All approaches had lower average silhouette scores compared to CLR-normalized data (CLR-normalized *ss* = 0.488; ComBat *ss* = 0.188 ComBat-Seq *ss* = 0.11; RUVg *ss* = 0.16; RUV-III-NB *ss* = 0.12; RUVs *ss* = 0.32), suggesting successful removal of storage condition effects. Overall, correction using RUV-III-NB and ComBat-Seq yielded the lowest average silhouette scores for clustering by storage conditions within this dataset, indicating their robustness in removing unwanted variations from the major source of technical variation. Figure 3A,B confirm the successful removal of storage effects using RUV-III-NB as the clustering based on storage conditions and library preparation kits previously apparent in CLR-normalized data (Fig. 2B) were no longer visible after correction with RUV-III-NB.

We also calculated the RLE metric score based on the within-pig variations of the RLE medians and interquartile ranges (see “Performance metrics calculation” subsection in “Materials and methods” for details), where a higher value of the metric is associated with better performance. RUVg garnered the best average RLE metric across different strategies for defining negative control taxa (Ω_*RLE*_ score = 8.64), followed by RUV-III-NB (Ω_*RLE*_ score = 6.95) (Fig. 3D). Both ComBat-based methods lagged behind for this metric as ComBat (Ω_*RLE*_ score = 4.11) performed comparably to CLR-normalized data (Ω_*RLE*_ score = 3.98) and ComBat-seq (Ω_*RLE*_ score = 2.94) scored even lower than CLR-normalized data. This suggests that RUV-based methods perform better when it comes to removing the effects of overall (major and minor sources of) unwanted variations in microbiome data than ComBat-based methods. Figure 3C demonstrates the success of RUV-III-NB correction as the within-pig RLE medians and interquartile ranges become more consistent compared to CLR-normalized data (Fig. 2C).

We further demonstrated the effect of successfully removing unwanted variations on bacterial abundance. To this end, we performed differential abundance analysis using edgeR^31,32^ between the different storage conditions, in which only spiked P1 samples were used. Since these samples come from the same pig, we expect little-to-no differentially abundant taxa (π_0_ = 1) when unwanted variations are successfully removed. We use Storey’s approach for estimating the proportion of null (non-differentially abundant) taxa (π_0_) based on the p-value distribution from the differential abundance analysis^33^. When comparing averages, ComBat-Seq returned the best proportion of null out of all the methods (π_0_ = 0.905) (Fig. 3E). Correction using RUV-III-NB resulted in the highest π_0_ compared to the other RUV methods (π_0_ = 0.825), followed by RUVs (π_0_ = 0.79). It is important to note that individual performances of each method using the different versions of negative control taxa result in a different rank of performances, which is detailed (in the *Supplementing control features with empirical negative control taxa is preferred when using RUV-based methods* section) below. Taken together, these observations suggest that RUV-III-NB performed the most consistently robust compared to the other methods in removing unwanted variations.

### RUV-III-NB preserves individual-specific biological signals when removing unwanted variations

**Figure 4:**
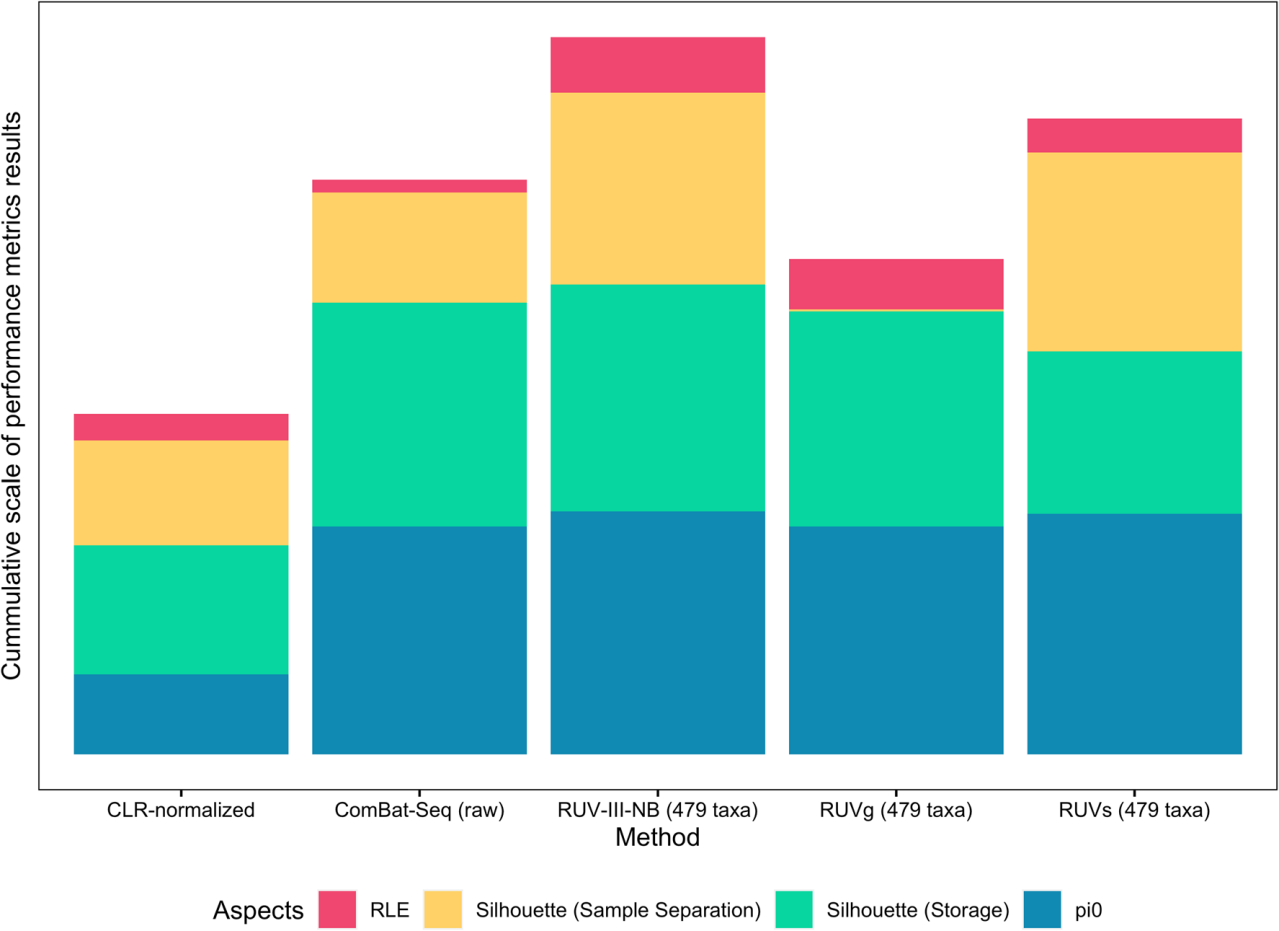
Summarized plot showing the performances of all the methods used to remove unwanted variations in pig faecal samples. For methods utilizing control features (RUVg, RUVs, and RUV-III-NB), we only picked the attempts using combination control taxa (479 taxa) as they demonstrated overall best performances within each method. For every method represented by the stacked bar, each color represents the performance of each method in each specific metric (as previously shown in Fig. 3); for visual uniformity, a taller stacked bar represents overall better performance. For RLE, the value represented was taken from 1-overall canonical correlation of the RLE metrics and known unwanted technical variations; for silhouette (storage), the value was taken from 1- average width of silhouette statistics score for clustering by storage conditions.

To assess how well correction methods preserve biological differences between pigs, we calculated silhouette score statistics for clustering by pigs, after removal of unwanted variations, a metric describing how well samples from different biological conditions (here, pigs P1 and P2) separate after clustering, based on how close each point in one cluster is to points in the neighboring clusters; in this case, high value indicates precise clustering and separation of samples from different pigs and, whereas low value suggests otherwise. RUV-III-NB had the highest average silhouette score (ss = 0.75), followed by RUVs (ss = 0.648) and ComBat-Seq (ss = 0.437) (Fig. 3F). Interestingly, RUVg yielded the lowest average score overall (ss = − 0.009). This is clearly demonstrated in the PCA plots before and after correction, in which separation of P1 and P2 samples are evident in PC1 and PC2 coordinates even before any correction (Fig. S3), with RUVg the only methods that removes this separation. The discrimination between individual metagenomic profiles is apparent in CLR-normalized data (Fig. 2A) and is retained after RUV-III-NB correction (Fig. 3A), but not after using RUVg (Fig. S3). Out of all benchmarked methods, RUVg is the only one that do not require users to specify replicate information, i.e. which samples belonging to which pig. These results suggest that replicate information is necessary when correcting unwanted variations to avoid over-correction. On the other hand, it is worth noting that out of all methods that use replicate information, RUV-III-NB has considerable edge when assessed over multiple performance metrics (Fig. 4).

To further assess RUV-III-NB’s ability in preserving biological differences when analyzing samples with a higher degree of diversity, we performed additional analysis using RUV-III-NB on an external dataset of 39 samples taken from 11 different water treatment plants using rapid sand filters (RSFs)^34^. As the dataset did not include any spike-in taxa, we identified empirical negative control taxa, which are taxa that are constantly abundant across samples within a dataset (see “Strategies for identification of empirical negative controls” section in “Materials and methods”). Using solely 2112 empirical negative control taxa, RUV-III-NB was able to remove unwanted technical variations while still retaining true biological signals of each replicate group and primary source of microbial diversity; clustering based on replicate groups is improved (CLR-normalized *ss* = 0.20; RUV-III-NB *ss* = 0.569) and separation based on water source slightly improved (CLR-normalized *ss* = 0.457; RUV-III-NB *ss* = 0.533) after correction (Fig. S4).

### Supplementing control features with empirical negative control taxa is preferred when using RUV-based methods

We have demonstrated that on average, RUV-based methods have the edge when it comes to removing unwanted variations from microbiome data with RUV-III-NB performing the most consistently robust across the board. However, within each RUV-based method, their performance varies when different sets of negative control taxa are used (Figs. 3D–F, and S1). Overall, the performance is worst when only spike-ins taxa are used as negative control and the best when the spike-ins taxa are supplemented using empirical negative control taxa. Due to the nature of the original experiment^27^, our spike-ins taxa are also naturally present in biological samples. We investigate if this could be the reason behind the failure of spike-ins taxa in removing unwanted variation. For each spike-ins taxon, we compared the average counts per million (CPM) between the spiked and unspiked samples. We found that the average CPM of spike-ins taxa in the spiked samples are at least 46 times that of the unspiked samples (Tables S1–S3). This indicates that among the spike-in taxa, the proportion of sequencing count that comes from a naturally-occurring state is relatively low. Further, we also performed sensitivity analysis by re-running RUV-III-NB algorithm excluding the two spike-ins taxa for which the proportion of counts from naturally-occurring states are the highest (*Saccharomyces cerevisiae* S288C and *Cryptosporidium parvum* Iowa II) and found the estimated unwanted factors are not sensitive to the exclusion of these two taxa (Table S4). Taken together, this suggests that the failure of spike-ins taxa is likely not due to the naturally occurring characteristics of the taxa but because its small number of features cannot fully capture the complexity of unwanted variation in microbiome data. When we supplement the negative control taxa using empirical negative control taxa, we observed a significant improvement across different performance metrics.

Taken together, our results suggest that utilizing empirical negative control taxa on top of spike-in taxa as control features should yield the most optimal removal of unwanted variations when using RUV-based methods. When no spike-ins taxa are available, using solely empirical negative control taxa still result in improved corrective performance, which was also demonstrated by our additional analysis on the RSF samples (Fig. S4). Given the importance of empirical negative control taxa, we provide a general framework for identifying these taxa in the “Strategies for identification of empirical negative controls” section below.

### Unwanted variations correlate with known sources of technical variations, and mostly affect highly abundant taxa

**Figure 5:**
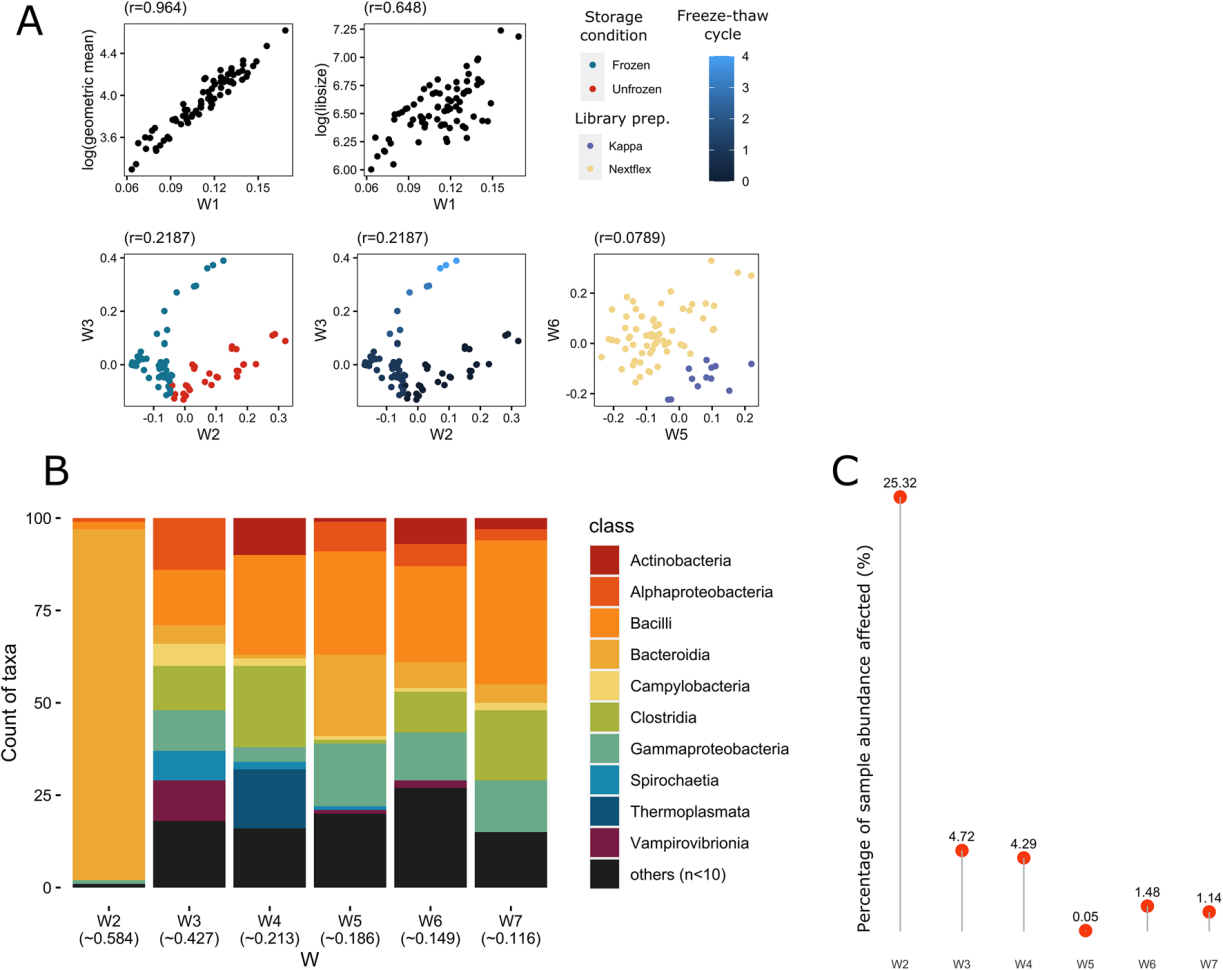
**(A)** Factors of estimated unwanted variations (Ws) from RUV-III-NB correlated with known batch information: unwanted factor 1 (W1) correlated with log library size and log geometric mean, W2 correlated with storage conditions, W3 correlated with freeze–thaw cycles, and both W4 & W5 correlated with library preparation kits. **(B)** The top 100 microbial taxa most affected by each W colored by Class, ranked based on Veall-Zimmermann pseudo R-squared (mean listed under every W). Classes with less than two microbial taxa were merged as “others”. **(C)** The proportion of a sample’s total abundance affected by the top 100 microbial taxa for each W.

RUV-based methods do not assume that users have any knowledge about the source of the unwanted variation. Instead, these unwanted factors are estimated empirically from the data using the negative control taxa. We have seen how RUV-III-B manages to remove unwanted variation from the current dataset. To further understand the possible origin of these unwanted variations, we correlate the estimated unwanted factors (**W)** with known experimental factors in the current dataset. The estimated primary unwanted factor (W1) correlated highly with log geometric mean of sequencing count (r = 0.964), revealing that sequencing depth, as to be expected, is the major source of unwanted variation in the dataset. Several known experimental factors were also found to correlate well with other unwanted factors: we found storage conditions and freeze–thaw cycles correlating with W2 (r = 0.770) and W3 (r = 0.782), respectively (Fig. 5A). Differences in the library preparation kit used were also found to moderately correlate with W5 (r = − 0.522) and W6 (r = 0.500). This supports our initial observation of storage conditions being the primary source of unwanted variations in our dataset once the sequencing depth effect is removed using CLR-normalization. This also highlights RUV-III-NB’s ability to capture unwanted variations originating from differences in experimental techniques, allowing us to correct the data without prior knowledge of the source of the unwanted variations.

### Technical variations do not affect different taxa uniformly.

Through the estimated unwanted variations information from RUV-III-NB, we also discovered that not every taxon is affected similarly by variations in technical procedures, and that some taxa are more sensitive to varying experimental conditions than others. Using a negative binomial generalized linear model, we estimate how much each of the unwanted factors affects each taxon in our dataset (see “Quantifying relative contributions of unwanted factors” section in “Materials and methods”). Microbial taxa belonging to the *Bacteroidia* class were found to be the most affected by W2, which correlated to storage conditions of the samples (Fig. 5B). For W3, which correlates with freeze–thaw cycles, members of the *Bacilli* class make up most of the top 100 most affected taxa. Members of the *Bacilli* class also make up most of the top 100 taxa most affected by W5 and W6 which correlates with library preparation kits. Members of *Clostridia* class were most prominent among the top 100 taxa affected by W4 and W7;. Incidentally, *Bacteroidia, Bacilli,* and *Clostridia* are the most abundant classes in the dataset, with 2153 species belonging to *Bacteroidia*, totaling over 656 million reads (62.8% of the total). To estimate the extent to which each of the detected unwanted factors affected our samples, we calculated the proportion of read counts belonging to the 100 most affected taxa, defined by the taxa with the highest pseudo-R^2^ for each unwanted factor (Fig. 5C). We found that storage conditions (whether samples were kept frozen or at room temperature before processing), represented by unwanted factor W2, affected up to 25.32% of read count—a notable amount considering only 100 out of 8453 taxa were taken into account (Fig. 5C). This further suggests that after library size, storage conditions are the primary source of unwanted variation in microbiome studies. Unwanted factors W3 and W4 contributed to 4.72% and 4.29% of overall sample abundance, respectively, whereas W5–W7 each contributed to under 2%.

## Discussion

The impact of technical variations in microbiome analysis is an important topic that has been explored numerous times, and past studies have reported on the best practices at every stage of sample processing^35^. However, our study is the first to quantify the contribution of different technical factors towards unwanted variations in a shotgun metagenomic sequencing dataset, and how to best remove them. To do so, we used RUV-III-NB to estimate the factors of unwanted variations, as well as compared its performance against other popular published methods.

### Inconsistent sample storage can be a large source of unwanted variations in microbiome studies

The variety of storage conditions—which encompass factors such as temperature, time, and storing method used—have been previously found to introduce considerable effects on microbial abundance^13,14,36,37^. The impact of different storage conditions is also not limited to microbiome studies, as Hickl et al. also reported significant differences in microbial protein identification between samples stored using flash freezing and RNAlater^38^. Our study does not aim to replicate and validate the findings of such studies, but to show how, if left unaddressed, the differences in sample storage conditions in microbiome studies could introduce significant changes in microbial abundance of abundant taxa which are potentially important for our understanding of particular conditions. The three most-affected bacterial classes in our study, namely *Bacteroidia*, *Clostridia* and *Bacilli*, are among the most dominant microbial classes in the mammalian gut^39–41^, therefore it is not surprising to see them strongly affected by technical variations. Both *Bacteroidia* and *Clostridia* classes in particular have been found to be significantly impacted by different storage methods in both metagenomic and metaproteomic data^38^. In clinical cases, the dysbiosis of members of the classes *Bacteroidia* and *Clostridia* have been linked to a wide variety of conditions, including T2D, Crohn’s disease, lupus, HIV infection, and major depressive disorders^41–46^. Hence, extra precautions are necessary in making sure the experimental design minimizes sources of unwanted variations as possible.

### Metagenomic sequencing depth also contributes to unwanted variations in microbiome studies

Aside from storage conditions, RUV-III-NB identified that geometric mean correlated with unwanted factor W1 in our study, which is unsurprising. In microbial metagenomics, sequencing depth typically impacts the robust classification of reads into genes or microbial taxa, and optimal depth to use depends on each study objectives^47,48^. However, when sequencing depth varies between samples, samples more deeply sequenced will tend to have higher count. This bias due to differences in sequencing depth is usually corrected using a normalization procedure. Normalization by library size, also known as TSS normalization, remains widely used in the field mostly due to conceptual simplicity that assumes average count to scale linearly with library size^49^. However, in our experiment we found that the first unwanted factor, W1 correlates much stronger with geometric mean of counts per sample than library size, suggesting that normalizing count by geometric mean (e.g., CLR transformation) rather than by library size (e.g. TSS) is potentially more effective in removing sequencing depth effect.

### RUV-III-NB is a robust and consistent microbiome batch correction method

Although the observation of best practice methods and preventive measures are always recommended when it comes to avoiding unwanted technical variations in any experimental setting, there are a few existing computational approaches to rectify variations due to unwanted effects after a dataset has been processed and sequenced. Here, we show that the effective removal of unwanted variations was still possible during downstream analysis, with different methods varying in efficiency.

ComBat-based methods rely on known batch information and might not be suitable in the realistic situation of multiple unwanted effects conflating with true biological signals. Nevertheless, when batch information is clearly defined, ComBat-based methods remove unwanted variations efficiently from our samples, but also impact the recovery of true biological signals separating individuals. In our work, ComBat-based methods were also unable to identify and adjust for unknown unwanted variations, which are expected to be common and unavoidable in practice. RUV methods avoid this hurdle by adjusting samples based on the variations of the control features.

One potential issue with RUV methods is that, unlike with RNA-seq data where housekeeping genes have been established^50,51^ and are routinely used to normalize expression and thus serve as excellent choice for control features, there is no obvious parallel in microbiome features, as taxa composition varies considerably across datasets. The choice of control features is especially crucial for the methods to work as intended; therefore, the presence of spike-ins is highly beneficial when utilizing RUV methods in microbiome studies. Ideally, spike-ins taxa need to be either not present or present with very low concentration (relative to the added concentrations from spike-ins) in the samples. For spike-ins, it is especially important to carefully curate them so they do not confound with biological taxa of interest or highly dominant taxa that occur naturally within the subject. In our experiment, the spike-ins are naturally present but their relative concentration is quite low. In this study, correction using only 8 spike-in control taxa removed unwanted variations to an extent, though using the additional empirical negative control taxa significantly improved the performance. The number of control features have an impact on the ability of RUV-based methods in removing unwanted variations. Higher number of control features are required to fully capture more complex unwanted variations. For example, in single cell RNA-seq experiments, we usually use 500–1000 control features^26^ We recommend that the number of spike-ins control taxa in microbiome needs to be at least similar to the 92 ERCC spike-ins frequently used in RNA-seq studies^52^. When spike-ins taxa are unavailable or are logistically hard to obtain, we recommend that researchers use empirical negative control taxa identified using our recommended strategies (see “Materials and methods”), as also demonstrated in our additional analysis with RSF samples (Fig. S4).

When comparing between RUV methods, we showed how inclusion of sample replicate information is important, as demonstrated by RUVg’s tendency to overcorrect and lose individual separation in the process. Both RUVs and RUV-III-NB utilize sample replicate information, and are therefore able to avoid overcorrection, yet the former performed less consistent across the performance tests. RUV-III-NB has an advantage over RUVs due to its direct modelling of mean–variance relationship of count data—a feature not shared by RUVs. While we have not tested the tool using microbial taxa abundance from marker gene-based methods such as the 16 s rRNA sequencing, it should in principle still be applicable with their output in count format. This study also demonstrated RUV-III-NB’s advantage in being able to adjust for unwanted variations directly based on the microbial counts after taxa classification without having to perform an additional step of gene-level annotation, which is necessary when utilizing universal phylogenetic marker genes for normalization^53^. Note that the application of RUV-III-NB for metagenomic data is intended to be performed after taxonomic classification, therefore it does not affect any early step of MAGs reconstruction, and we do not recommend using the tool on contig counts.

### Recommended strategy for removing unwanted variation from microbiome data

As demonstrated in our results, using both technical replicates and negative control features are essential in removing unwanted variations using in-silico approaches such as RUV-III-NB. In metagenomic studies, negative control features should be taxa that are consistent in abundance across samples in different groups and/or treatments, which we could control during experimental design through the use of spike-ins. Though not demonstrated in our dataset due to resource limitations, we recognize that the most ideal choice for spike-ins would be to use genetic fragments that are completely distinguishable from natural species, such as using synthetic DNA spike-ins^54^. Yet, we demonstrated that utilizing naturally occurring taxa as control features still work as long as they are not dominantly abundant within the subject.

Additionally, we also demonstrated the benefit of utilizing features identified in-silico as *empirical* negative controls, which may be the only option for datasets that have already been sequenced without spike-ins. Using the pig faecal samples dataset, we were able to identify the empirical negative control taxa through differential abundance analysis between spiked and unspiked samples per replicate group, then take an overlap of the least differentially abundant taxa across the groups. For datasets without spike-ins, this could be done by running differential abundance analysis between samples within the same replicate group randomly assigned into two different hypothetical groups, from which an overlap could be taken from the least differentially abundant taxa across the groups. When the number of replicate group is limited, or for a much stricter set of empirical taxa, running the differential abundance analysis could then be repeated by using random permutations of the hypothetical groups within a replicate group. Using the additional RSF water metagenomic samples, we were able to demonstrate the feasibility of obtaining empirical negative control taxa this way, as we identified 2112 empirical negative control taxa and used them to normalize the samples with RUV-III-NB without the need of spike-in taxa. This additional analysis also highlights RUV-III-NB’s versatility, as its corrective performance remains robust on a dataset with a different type of sample (environmental vs organismic) and low number of replicates.

For the specific purpose of this study, a variety of treatments were done to the samples deliberately to capture the breadth of unwanted technical variations that may exist. Hence, we did not run any contaminant removal procedure as it may minimize the variations we deliberately aimed to represent. On top of that, our usage of spike-ins may be identified as contaminants by the tool despite them being introduced to the samples by design. Although their combined performance has not been tested, we do see RUV-III-NB being compatible with contaminant removal procedures such as *decontam*^55^. As RUV-III-NB focuses more on adjusting the data based on negative control features, the absence of more contaminants should only improve its performance, though extra caution is crucial to ensure the spike-ins are retained when included.

## Conclusions

In this study, we have shown how technical variations—specifically storage conditions, freeze–thaw cycle and library preparation kits—may introduce unwanted variations in microbiome data, affecting the observed abundances of important and dominant microbial taxa. Minimizing the possibility of introducing unwanted variations by limiting the presence of ‘batches’ and utilizing consistent storage conditions, library preparation kits and equipment are highly suggested. We did not examine differences between sequencing platforms in this study, but we believe sequencing platforms could also be a source of unwanted variations, especially where the data comes from very different platforms (e.g., PacBio and Sequel2). We finally show that for existing datasets, post-processing corrective measures can still be performed in silico to remove unwanted variations stemming from variations in experimental techniques, and we suggest the use of *RUV-III-NB* as a consistent and robust method.

## Materials and methods

### Sample processing, library preparation, and sequencing conditions

The fecal samples investigated in this study originated from two individual pigs (P1 and P2) collected right after defecation from two different conventional pig farms in Denmark as previously described^27,56^. Each individual pig faecal sample was homogenized with a sterile wooden spatula and then separated into two large aliquots. One of the aliquots was spiked with a freshly prepared mock community consisting of 6 bacterial and 2 eukaryotic microorganisms, namely *Propionibacterium freudenreichii* DSM 20271, *Bacteroides fragilis* NCTC 9343, *Staphylococcus aureus* NCTC 8325*, Fusobacterium nucleatum* ATCC 25586*, Escherichia coli* ATCC 25922*, Salmonella* Typhimurium str. ATCC 14028S*, Cryptosporidium parvum* IOWA II isolate, and *Saccharomyces cerevisiae* S288C; the details on the quantity and how the spike-ins were added are described the Supplementary Materials of a previous study^27^*.* The spiked fecal samples were homogenized using a sterile wooden spatula, and small aliquots for each sample storage condition were prepared in Eppendorf tubes for both, the spiked and unspiked aliquots. DNA was isolated immediately from the aliquots for the initial time point (storage for 0 h) using a modified QIAamp Fast DNA Stool Mini Kit (Qiagen) protocol with an initial bead beating step (MoBio garnet beads)^57^. The remaining aliquots were stored at different storage conditions comprising different temperature and time combinations (Fig. 1A). Samples were stored at 22 °C, 5 °C, − 20 °C, and − 80 °C for several hours (days) (16 h (0.67 days), 40 h (1.6 days), 64 h (2.6 days), 88 h (3.6 days)) and for the temperatures − 20 °C and − 80 °C also for months and up to one year (4 m, 8 m, 12 m). Aliquots stored for 40 h and 88 h, as well as a subset of 64 h, also underwent 2–4 freeze–thaw cycles. All fecal samples underwent the same DNA isolation method (see above)^27,57^ prior to library preparation.

Three different library preparation kits (the PCR-free NEXTflex and KAPA, as well as Nextera) and two sequencing platforms (HiSeq 4000 and NextSeq 500) were used in the study, although the spiked samples were sequenced exclusively using HiSeq 4000^27,56^. All samples were sequenced paired-end with a read length of 150 bp. Two to three technical replicates were performed for each treatment combination.

Together, a total of 184 different samples spread across 60 different replicate groups were acquired, encompassing 21 specific combinations of storage conditions, spiking status, library preparation kit used, as well as thawing cycles. The raw reads are deposited at the European Nucleotide Archive (ENA) (Project acc.: PRJEB31650).

### Quality control and taxonomic classification

Quality control of sequencing files was done following DTU Food’s in-house pipeline, FoodQCpipeline (https://bitbucket.org/RolfKaas/foodqcpipeline), in which BBMap’s bbduk2 (v38.71, https://jgi.doe.gov/data-and-tools/bbtools/) was used to trim reads with a length of at least 50 bp, Phred score of at least 20, and also remove a custom list of Illumina adapters. FastQC (v0.11.8) was also applied to the files before and after trimming to assess the quality of the reads^58^.

Taxonomic classification was done with Kraken2 (v2.0.9)^59^, followed by taxa abundance re-estimation using bracken (v2.5)^60^ at species level with read length of 150 and minimum taxa threshold of 1. A custom genomic index was used for the classification based on GTDB release 89^61,62^, which includes 23,458 bacterial and 1,248 archaeal species. To capture the eukaryotic spike-in counts, all samples were mapped to a custom reference containing the genomes of the 8 spike-in taxa using bowtie2^63^, then counts per sample were calculated using featureCounts^64^. Counts of the 6 bacterial spike-ins from bowtie2 were found comparable with the Kraken2 results (Table S5). The eukaryotic spike-in counts from bowtie2 were included and Kraken2 results for all bacterial counts were kept for further analyses. After merging individual reports, taxa filtering was done based on counts per million (CPM) > 4 in at least 15% of all samples. A total of 8,453 taxa in the pig faecal samples dataset were included for downstream analysis.

For the additional analysis on RSF water metagenomic dataset^34^, the same quality control and taxonomic classification procedures were applied.

### RUV-III-NB method

The novel RUV-III-NB method^26^ (https://github.com/limfuxing/ruvIIInb/) extends the RUV-III (Removing Unwanted Variation-IIII) method that was previously developed for array-based gene expression data^26,65^. Instead of using linear model, it uses a Negative Binomial-based generalized linear model (GLM) with log link function to model the effect of wanted biological signals and unwanted variations on the sequencing count at taxon-level. Briefly, the count for taxon i in sample j is modelled as Negative Binomial random variable with mean µ_ij_ and dispersion parameter φ_i_. This mean parameter is influenced by biological and unwanted factors through the log-linear model

$${\text{log }}\upmu_{{{\text{ij}}}} = {\text{z}}_{{\text{i}}} + {\mathbf{M}}\upbeta_{{\text{i}}} + {\mathbf{W}}\upalpha_{{\text{i}}}$$

where ζ_i_ is the intercept parameter, **M** (N x m) is the matrix that contain (technical) replicates information with m_pq_ = 1 if sample p is a (technical) replicate of individual q, β_i_ (m × 1) is the biological regression coefficient that governs how the *m* individuals differ biologically in terms of taxon I, **W** (N x k) is the *unknown* k-dimensional unwanted factors that generates the unwanted variation and α_i_ (k × 1) is the regression coefficient that governs how the unwanted factors affect taxon i. The RUV-III-NB algorithm uses iterative reweighted least squares (IRLS) to estimate parameters of interest: **W,** ζ_i_, β_i_, α_i_ and φ_i_. In particular, we use the information contained in the replicate matrix **M** to estimate α_i_ and given α_i_ estimate we use negative control feature to estimate **W.** Readers interested in more detailed about the RUV-III-NB model, methodology and implementation can refer to^26^ Note that we do not assume that the unwanted factors **W** are known. Instead, these are estimate empirically from the data through the variations inferred from the negative control features. This is a unique feature of RUV-based methods and can be considered as one of their comparative advantages because in reality, we often only have partial knowledge of the unwanted factors. But because these unwanted factors are estimated empirically from the data, we often correlate the estimated unwanted factors with some known experimental factors to provide a more tangible interpretation to the estimated **W**. Note that each dimension of the estimated unwanted factors does not need to have a high correlation with a known experimental factor, but a high correlation provides an informal validation that the dimension ‘represents’ the particular experimental factor and thus the experimental factor does contribute to the unwanted variations in the data. Each dimension of **W** explains a different amount of unwanted variation with the first dimension (referred to as W1) explains the most amount and successive dimensions (e.g. W2, W3, W4) explains less and less amount.

Once the parameters have been estimated, RUV-III-NB returns the percentile-invariant adjusted count (PAC) to represent the sequencing count matrix that has been adjusted or corrected for the unwanted variations. The percentile-invariant adjusted count was calculated based on a modification version of the randomized quantile residual method^66^.

### Benchmarking methods for removing unwanted variation

We compare the effectiveness of the following methods for removing unwanted variation: ComBat (sva v3.42.0)^19,29^, ComBat-Seq (sva v3.42.0)^30^, RUVg (RUVSeq v1.28.0)^20^, RUVs (RUVSeq v1.28.0)^20^, and RUV-III-NB (v0.7.6.2)^26^. For a fair comparison, analyses were done only on spiked samples, though empirical negative control taxa were also identified and tested for RUV-based methods to demonstrate their performances without known spike-in taxa. ComBat and ComBat-Seq both utilize known batch variable to remove unwanted effects from a dataset, though the former allows normalized and/or log-transformed matrix as input, whereas the latter requires raw integer count matrix as input^29,30^. RUVg, RUVs, and RUV-III-NB all utilize control features in estimating unwanted factors. Both RUVg and RUVs assume an underlying Normal model and take normalized and/or log-transformed matrix as input, while RUV-III-NB only accepts integer count matrix; though both RUVs and RUV-III-NB require sample replicate information^20^. Out of all the methods, only ComBat-Seq and RUV-III-NB deal directly with the mean–variance relationship in the count data by using NB distribution. We performed Aitchison’s centered log-ratio (CLR) transformation on raw data prior to ComBat correction^67–69^. Prior to RUVg and RUVs, CLR transformation was also performed. For RUV methods, we set $k$—which represents the number of unwanted factors estimated from the data—to 7 as it is the highest possible *k* when using the smallest set of negative control features, i.e. the spike-ins taxa. We do not determine there are only 7 unwanted factors but for the sake of simplicity and conciseness in our reporting, we have decided to estimate only seven unwanted factors that explained the highest amount of variance in the data. Setting a higher k would make the comparison of results obtained using different set of negative control features meaningless. For RUV-III-NB, we also set the parameters *lambda.a* = 0.01 and *lambda.b* = 5.

### Strategies for identification of empirical negative controls

In our main analyses with pig faecal samples, we curated a set of empirical control taxa to be used as control features in RUV methods in addition to the 8 spike-in taxa. To identify empirical negative controls, a partial knowledge of the main source of unwanted variation is helpful, although not compulsory. Our procedure for identifying empirical negative controls used for the pig facial sample dataset is as follows:

1. Specify the biological factors of interest in the study. In our case, this is the individual pig identity.
2. Perform differential abundance analysis between samples with different biological factors of interest using edgeR package^31,32^. In our case, this involves comparison between P1 and P2. If users have knowledge about the main source of unwanted variation, the comparison between P1 and P2 can be adjusted by including variable(s) that represent the main source of unwanted variation, e.g. storage condition in the model. In our case, we assume no knowledge of this variable and perform the differential abundance analysis between P1 and P2 unadjusted.
3. Take ~ 1000 taxa with the highest p-value from the analysis in Step 2 as *empirical negative control* taxa. These taxa are chosen because they have the least biological signals and therefore when used as negative controls are the least likely to remove biological signals.
4. The list of taxa from Step 3 can be refined further if spiked and unspiked samples from the same biological condition are available. This is true for our dataset because we have spiked and unspiked samples from each pig. To refine the list, we identify taxa that not only contain the least amount of biology but are also the least affected by the spiking process. The following steps can be used to achieve that:
  - For each biological condition (each pig), perform differential abundance analysis comparing spiked and unspiked samples. If users have knowledge about the main source of unwanted variation, e.g. storage condition, the differential abundance analysis can be stratified by these factors.
  - From each biological condition, obtain the taxa least affected by spiking process. In our case, we exclude the top 15% taxa based on p-values from step a above.
5. We perform step 4b for all biological conditions and obtain the intersection of taxa that consistently appear to be least differentially abundant based on p-values for all biological conditions (all pigs).
6. Take the intersection between taxa that we obtain from steps 3 and 5 as empirical negative control taxa.

In our dataset, using the above procedure we identified 471 empirical negative control taxa. The performances of RUV-based methods were assessed while using either solely spike-in taxa (8 taxa), solely empirical taxa (471 taxa), or the combination of both as control features (479 taxa).

In our additional analysis with RSF water metagenomic samples^34^, the dataset contained no spike-ins and had higher variability, as the 39 samples were spread across 11 different replicate groups (1–4 replicates each) representing different water treatment plants. Due to the low number of replicates per group and a high number of biological sources, acquiring the empirical negative control taxa required a slightly different approach. The steps to curate empirical negative control taxa from this dataset, and our recommended procedure for identifying empirical negative controls with no spike-in taxa, are as follows:

1. Specify the factors of interest in the study. For this dataset, these are the water source and the filter material of the RSFs from each water treatment plant.
2. Based on a factor of interest, randomly assign the samples into two hypothetical groups with a balanced number of samples per variable. For example:
  - Regarding the water source, samples were divided into two groups with a balanced number of *Surface* and *Ground* samples.
  - Regarding filter material, samples were divided into two groups with a balanced number of *Sand* and *Carbon* samples.
3. Perform differential abundance analysis using edgeR package^31,32^ between the two hypothetical groups, and take the *n* least significant taxa from the results. In our case, considering the low amount of significantly differentially abundant taxa and the multiple overlaps from random permutations to be done in the next steps, we took > 75% of the taxa with the highest p-value from the analysis.
4. Repeat steps 2 and 3 to get randomized makeup of the hypothetical groups via random permutations. In our case, we repeated it 10 times per factor of interest.
5. Take the intersection between the lists of least significant taxa from all the differential abundance analysis permutations in step 4. In our case, we overlapped the results from the 10 attempts.
6. Repeat step 5 with a different factor of interest. In our case, after getting an overlapped list of least significant taxa between hypothetical groups based on water source, we repeated the steps based on filter material. At the end of this step, we ended up with two lists of taxa, each one basing its hypothetical grouping on a different factor of interest.
7. Take the intersection between lists of taxa obtained from step 6. These are your empirical negative control taxa.

Using the above procedure, we identified 2,112 empirical negative control taxa for the RSF water metagenomic samples.

### Performance metrics calculation

#### Relative log expression (RLE)

RLE plots visualize the presence of unwanted variations by calculating the deviations of from the median of each feature, in this case microbial taxa^28^. For RUV-III-NB, the log of percentile-invariant adjusted count (PAC) was used as the adjusted data matrix for the visualizations. To compare the RLE plots between methods, we calculated a metric capturing the average within-individual variance of RLE medians (V_RLE-med_) and the average variance of RLE interquartile range (V_RLE-IQR_), and use Ω_RLE_ = − log(V_RLE-med_ + V_RLE-IQR_) to represent the overall quality of the RLE. Because we expect a good normalization would result in RLE with small V_RLE-med_ and V_RLE-IQR_, a larger Ω_RLE_ is associated with better performance.

We also calculated NCC_RLE_ = 1-total canonical correlation^70^ of the RLE medians and IQRs and the known sources of unwanted variations as an additional metric. A large NCC_RLE_ value indicates a large remaining correlation between the normalized data and the unwanted factors, indicating failure in normalization.

#### Silhouette statistics based on principal component (PC) scores)

We calculated the PC score using Principal Component Analyses and used the first four PC scores to calculate the silhouette statistics using the ‘cluster’ package^71^ (version 2.1.0) to assess two different aspects:

- Preservation of biological signals, in which we calculated the silhouette score for clustering by individual pigs (P1 and P2) samples and higher scores signify better preservation.
- Removal of Unwanted variations, in which we calculated the silhouette scores for clustering by an experimental factor (e.g., storage condition) within each pig group, with lower scores signifying better removal.

#### Differential abundance analysis

We also performed an additional specificity assessment for the methods through differential abundance analysis using edgeR^31,32^ on samples from the same source (P1) between frozen and unfrozen storage conditions.

- ComBat is excluded from this analysis as it only produces a transformed adjusted data matrix and not the estimated factors of unwanted variations necessary for covariates in differential abundance analysis.
- Corrected count from ComBat-Seq was directly used as input for the analysis using edgeR with frozen/unfrozen storage condition as covariates.
- For RUV methods, original counts were used as depedent variable with the frozen/unfrozen storage condition along with the estimated unwanted factors (W) used as covariates.

We set the significance threshold to those with p-value < 0.05 after FDR correction and with absolute log-fold change > 1. The proportion of null taxa was estimated using the qvalue function in the qvalue Bioconductor package.

### Quantifying relative contribution from unwanted factors

We used RUV-III-NB to quantify unwanted factors in the data and analyze their relative contribution towards microbial taxa abundance. Hence, for our main analysis only spiked samples were used as the approach requires control features as input, which in our case included the 8 spike-in taxa and an additional set of 471 empirically constant taxa explained in the previous section. We set the same number of $k$=7 as stated in the previous section for consistency. We then used a negative binomial generalized linear model (*glm.nb*) and model the sequencing count of each microbial taxa as a function of (progressively) cumulative unwanted factors. We calculated the Pseudo‐R^2^ from each model using the PseudoR2 function from the ‘DescTools’ R package (version 0.99.38)^72^ with Veall-Zimmermann correction^73^, as it is among the closest approximations to ordinary least square R^2^. The contribution of each individual unwanted factor (W) towards each microbial taxon is represented by the difference in pseudo-R^2^ between the cumulative nth and n − 1th models (i.e. contribution of W3 was calculated by subtracting the pseudo-R^2^ accounting for unwanted factors W1 + W2 + W3 with the value accounting for unwanted factors W1 + W2). To see the effect of known technical variations on microbial abundance, we then took the top 100 affected microbial taxa by each of the individual factor of unwanted variations, ranked based on their pseudo-R^2^ and grouped based on taxonomic class. From the top 100 affected microbial taxa in each factor of unwanted variation, we calculated the proportion of their abundance in an average sample. For each of the most dominantly affected microbial classes, we also performed Wilcoxon signed rank test between storage conditions and Kruskal–Wallis test to compare the different freeze-thawing cycles and library preparation kits on log2-transformed, TSS-normalized data.

## Supplementary Information

Supplementary Information 1.

Supplementary Information 2.

Supplementary Information 3.

Supplementary Information 4.

## Data Availability

The raw sequence data was deposited at the European Nucleotide Archive (ENA) under accession number PRJEB31650.

## Abbreviations

CLR: Centered log-ratio
CoDA: Compositional data analysis
ENA: European Nucleotide Archive
GLM: Generalized linear model
IQR: Interquartile ranges
NB: Negative binomial
PC: Principal components
PCA: Principal components analysis
RLE: Relative log expression
ss: Silhouette statistics
T2D: Type 2 diabetes
TSS: Total sum scaling
