# Supplementary material for: Assessing and removing the effect of unwanted technical variations in microbiome data
Source: Sci Rep. 2022 Dec 23;12:22236. doi: 10.1038/s41598-022-26141-x (PMC9789116; doi:10.1038/s41598-022-26141-x)
Supplement: Supplementary file 1 — Supplementary Information 1. [file 41598_2022_26141_MOESM1_ESM.pdf]

Supplementary Figures

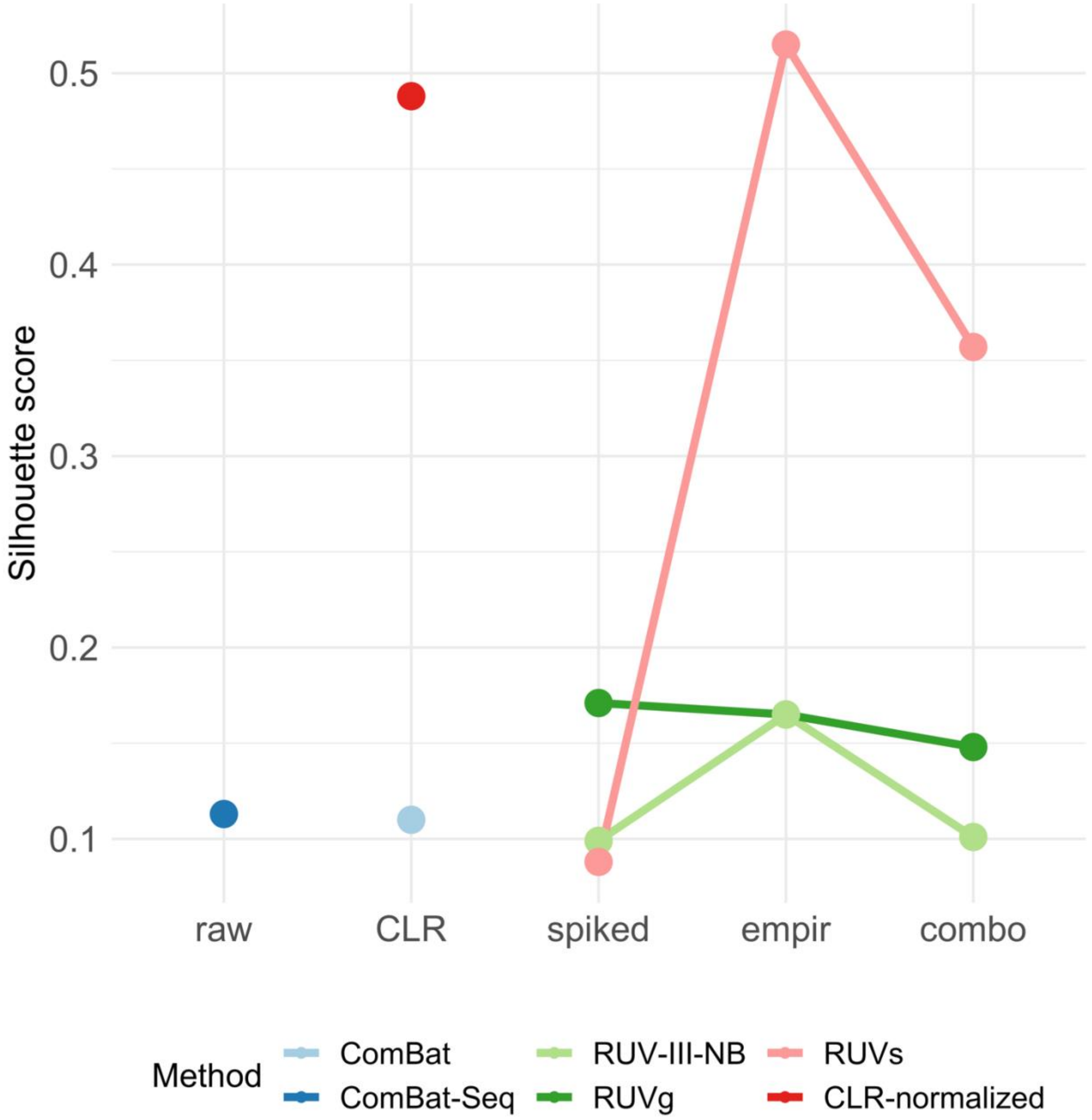

Figure S1: Comparison of correction method performances in removing unwanted variations from the main source (storage conditions). We calculated silhouette statistics based on the main principal components (PCs) of spiked samples, in which lower scores indicate poor clustering of batch variables, and therefore better removal of unwanted variations. With the exception of RUVs using only empirical control taxa (ss=0.51), all approaches had lower silhouette scores compared to CLR-normalized data (CLR-normalized ss=0.488; ComBat ss=0.11–0.26; ComBat-Seq ss=0.11; RUVg ss=0.14–0.17; RUV-III-NB ss=0.09–0.16; RUVs ss=0.08-0.51).

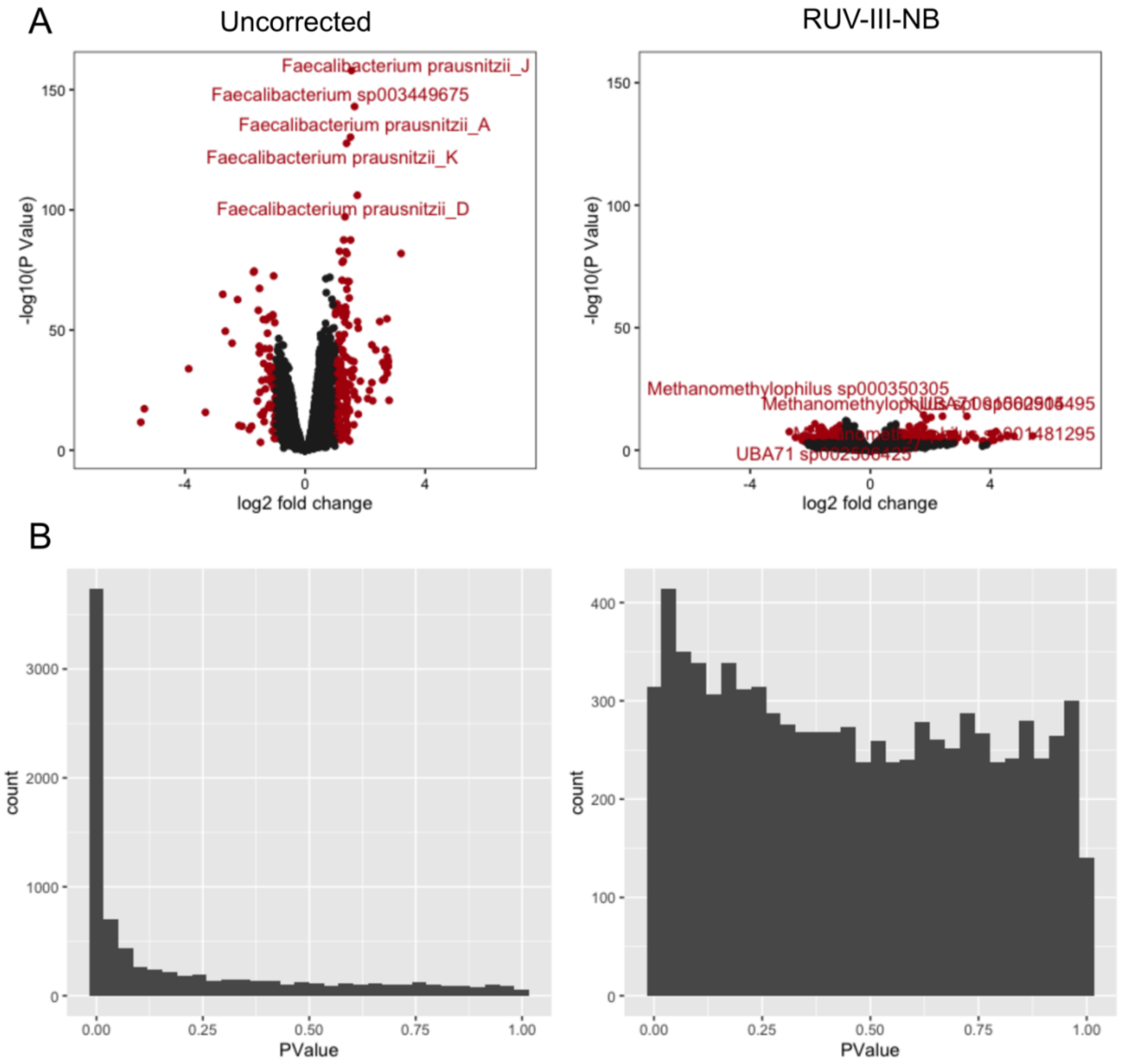

Figure S2: Results of differential abundance analysis of spiked pig 1 samples between storage conditions (frozen vs unfrozen), before (left) and after (right) correction using RUV-III-NB (with combination control features). Since the samples come from the same biological source, there should be little-to-no differences between the storage conditions. **(A)** volcano plot before correction shows over 200 significantly differentially abundant taxa (FDR<0.05, |log2(FC)|>1), with only 14 differentially abundant taxa after RUV-III-NB correction. **(B)** The distribution of p-values before correction is anti-conservative, noted by the peak on the left due to all the differentially abundant taxa. A more uniform p-value distribution can be seen after RUV-III-NB correction, suggesting minimum number of differentially abundant taxa.

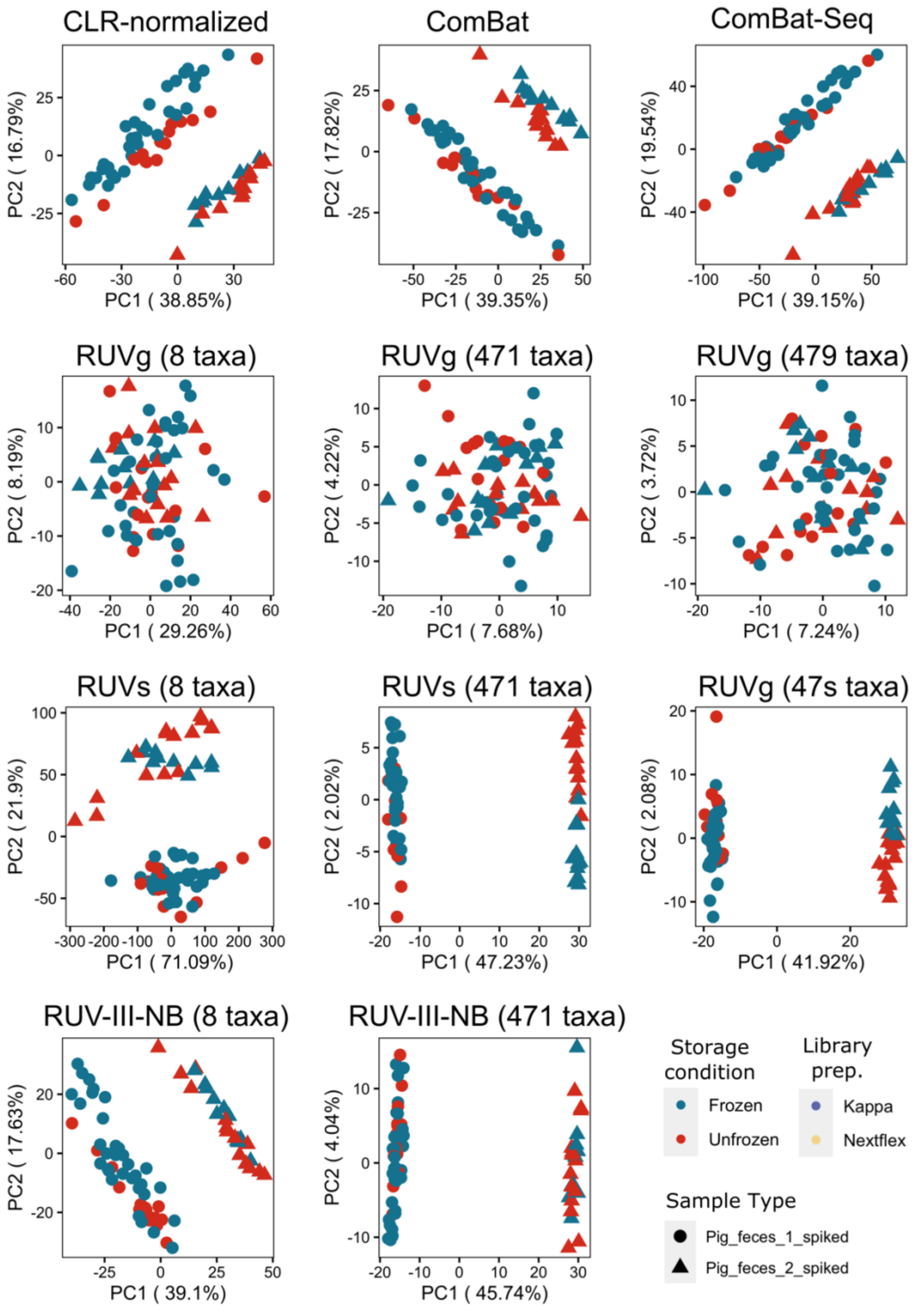

Figure S3: PCA plots showing the first two PCs (PC1-2) of all spiked samples after ComBat, ComBat-Seq, RUVg, RUVs, and RUV-III-NB correction (RUV-III-NB with combination negative control taxa shown previously in **Figure 3A**), highlighting how no matter which negative control taxa set was used, RUVg failed to retain individual biological information between pig 1 and pig 2 samples. Separation was seen in all other cases, even in CLR-normalized data (shown previously in **Figure 2A**).

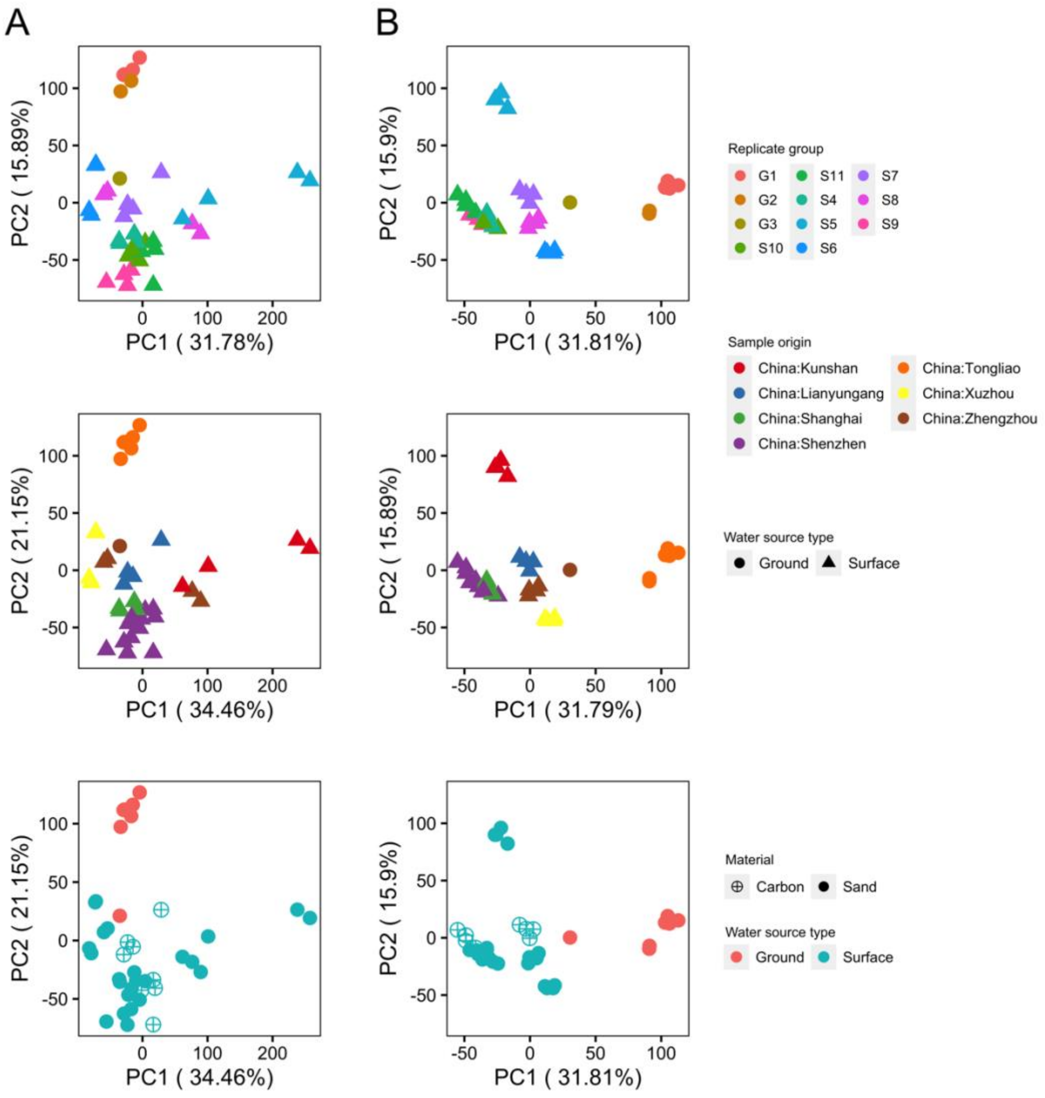

Figure S4: PCA plots showing the first two PCs (PC1-2) from the additional analysis on rapid sand filter (RSF) metagenomic samples, colored based on sample replicate groups, sample origin, and water source type. The study found that water source type was the primary source of microbial and functional diversity **(A)** CLR-normalized data showed separation based on water source type on PC2, with variations not clearly explained by either sample replicate groups, sample origin, water source type, or filter material being captured by PC1. Clustering based on sample replicate groups and sample origin could be seen. **(B)** After RUV-III-NB, slightly clearer separation based on water source type was captured in PC1, and clustering based on sample replicate groups and sample origin were more clearly defined while still retaining the individual differences of each replicate within the groups.

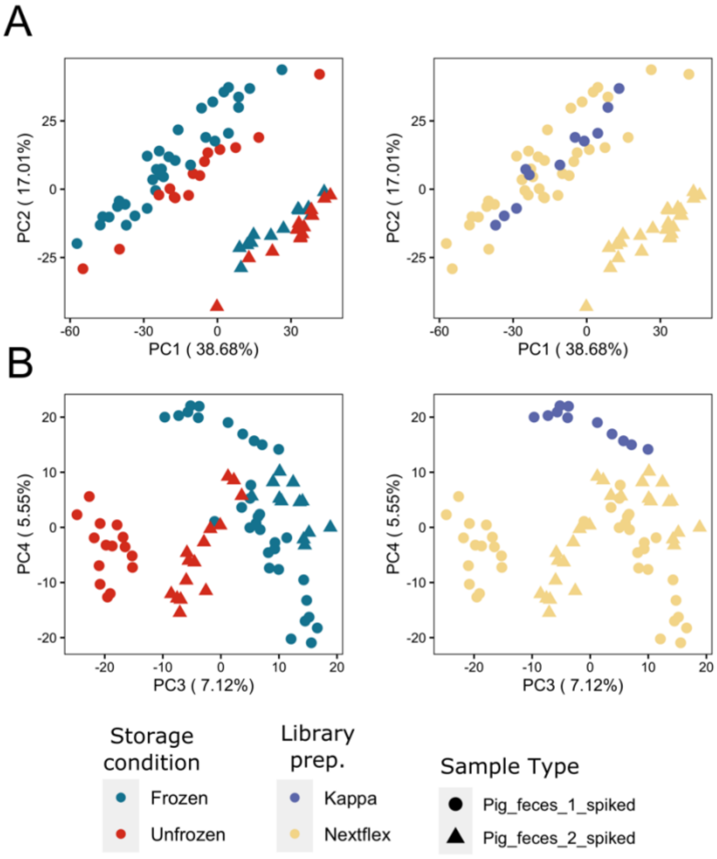

Figure S5: Visualizations of the additive log-ratio (ALR)-normalized data of all spiked samples: (A) Principal component analysis (PCA) plots showing sample clustering based on sample origin (pig1 vs pig 2) explained by PC1 and PC2, while (B) separation of samples based on storage conditions and library preparation kits used were explained by PC3 and PC4, respectively. Similar to using centered log-ratio (CLR) transformation (Figure 2), ALR transformation could not remove the impact of unwanted technical variations within the data.
